# Handgrip and sex-specific cardiometabolic risk factors in Hispanic/Latino migrant farmworkers

**DOI:** 10.1038/s41598-021-89138-y

**Published:** 2021-05-13

**Authors:** Anas Raed, Jessica Bilz, Miriam Cortez-Cooper, Lufei Young, Li Chen, Pamela Cromer, Haidong Zhu, Andrew Mazzoli, Samip Parikh, Jigar Bhagatwala, Yutong Dong, Zhuo Sun, Debbie Layman, Yanbin Dong

**Affiliations:** 1 Georgia Prevention Institute, Department of Medicine, Medical College of Georgia, Augusta University, 1120 15th Street, Augusta, GA 30912 USA; 2 Medical College of Georgia, Augusta University, Augusta, GA USA; 3 Rocky Mountain University of Health Professions, Provo, UT USA; 4 College of Nursing, Augusta University, Augusta, GA USA; 5 Respiratory Therapy Program, Augusta University, Augusta, GA USA; 6 Department of Anesthesiology and Perioperative Medicine, Medical College of Georgia, Augusta University, Augusta, GA USA; 7 Community Liaison Between Augusta University and Costa-Layman Farm, Augusta, GA USA

**Keywords:** Predictive markers, Cardiovascular diseases

## Abstract

Studies have suggested that handgrip strength might be a marker for cardiometabolic risk (CMR), but it has not been studied in Hispanic/Latino farmworker population. This study aimed to characterize absolute and relative handgrip strength in Hispanic/Latino farmworkers, and investigate the sex-specific association between handgrip strength and CMR factors. CMR factors and seated isometric absolute (the sum of both hands) and relative (absolute handgrip strength divided by body mass index) handgrip strengths were collected in 173 Hispanic/Latino farmworkers (mean age 35.1 ± 0.7 years; 49% female). The absolute and the relative handgrip strengths were 89.2 ± 1.8 kg, 3.3 ± 0.1 kg among males, and 56.5 ± 1.9 kg, 1.9 ± 0.1 kg among females, respectively. Age was correlated with absolute (*r* = − 0.17, *p* = 0.03) and relative handgrip strengths (*r* = − 0.28, *p* < 0.01). In males, absolute handgrip was related to triglycerides (*r* = − 0.25, *p* < 0.05), whereas relative handgrip was related to waist circumference (*r* = − 0.32, *p* < 0.01), waist/hip circumference ratio (*r* = − 0.36, *p* < 0.01), high-density lipoprotein (*r* = 0.24, *p* < 0.05), and triglycerides (*r* = − 0.35, *p* < 0.01). In females, absolute handgrip was related to fasting plasma glucose (*r* = − 0.28, *p* = 0.03), whereas relative handgrip was related to waist circumference (*r* = − 0.38, *p* < 0.01) and fasting plasma glucose (*r* = − 0.22, *p* < 0.05). Males had lower absolute handgrip strength when their triglycerides levels were at risk (*p* = 0.021), and lower relative handgrip strength when their plasma glucose (*p* = 0.034) and triglycerides (*p* = 0.002) levels were at risk. Females had lower relative handgrip strength when their plasma glucose (*p* = 0.001) and blood pressure (*p* = 0.004) were at risk. This study suggests that handgrip strength may be associated with sex-specific CMR factors in a Hispanic/Latino farmworker population.

## Introduction

Every year, approximately 4 million migrant farmworkers generate and support a $30 billion agriculture industry and associated economic vitality in the United States. Eighty percent of them are Hispanic/Latinos^1^. Despite the important role they play in the United States (US) economy, Hispanic/Latino migrant farmworkers represent one of the most socially vulnerable and disadvantaged workforces due to unstable working and living conditions and barriers to obtaining health services^2–4^. As a result of occupational hazards, and barriers to preventive healthcare services, there are increasing concerns of developing chronic diseases, such as cardiovascular disease (CVD) and its complications in this population. CVD, one of the leading causes of morbidity and mortality in the US^5^ and worldwide^6^, has especially high rates in racially and ethnically diverse populations, including Hispanic/Latinos^7^. Recent data also showed a high prevalence of cardiometabolic risks (CMR) among Hispanic/Latino populations, including diabetes, hypercholesterolemia, hypertension, and obesity^8^. Despite this increasing occurrence of CMR in the Hispanic/Latino population, very little evidence is available in the Hispanic/Latino migrant farmworkers. Previous studies showed 21.5% hypertension and 16.1% diabetes among farmworkers using self-report data^9,10^. Our group reported in a previous study that the prevalence of overweight/obesity, prehypertension/hypertension, and prediabetes/diabetes among Hispanic/Latino migrant farmworkers were 78.5%, 57.7%, and 36.7%, respectively^11^.

A number of epidemiological studies support the association between handgrip strength and cardiovascular health^12–14^. Handgrip strength, a surrogate marker for whole-body strength, is widely used to assess sarcopenia^15^. Handgrip is an emerging marker for CVD^16–18^ and diabetes^19,20^. Previous research has also indicated that early-onset obesity was related to decreased grip strength^21^. However, there are a few gaps in the literature. One study used race, including Hispanic, as a covariate, but did not focus on differences among the races in the relationship between handgrip and cardiovascular health biomarkers^16^. Mainous et al*.* showed that lower grip strength in both men and women is related to undiagnosed diabetes and hypertension in normal-weight individuals but not the general adult population^22^. In another study by the same research team, prediabetes was related to lower grip strength in men and women in whites but not Hispanics^23^. There is a study of handgrip strength in association with activities of daily living disability that was carried out among older Mexican Americans^24^. In addition to the absolute handgrip strength, relative handgrip strength consisting of the combination of handgrip strength and body mass index (BMI) was shown to diminish the confounding by body mass and accompanied health risks of elevated body weight^16^. There is a lack of research on the values of absolute and relative handgrip strength, and its relation to CMR in the younger Hispanic adult population in general, and particularly, Hispanic/Latino migrant farmworkers. The American Diabetes Association suggested that CMR factors include overweight/obese, high LDL cholesterol, low HDL cholesterol, high total cholesterol, high triglycerides, physical inactivity, high BP, high blood glucose, and smoking^25^. Thus, the aim of this study was first to characterize the values of absolute and relative handgrip strength in this population, followed by investigating the sex-specific association between handgrip strength and CMR factors of Hispanic/Latino farmworkers. We hypothesized that lower handgrip strength would be correlated with greater CMR factors.

## Methods

### Participants

This community outreach project was performed by the university and community partnership between Augusta University and CL Farm from 2013 to 2015. All Hispanic/Latino employees at the plant nursery in Trenton, South Carolina were invited to participate in this health-screening study. The consent form was translated into Spanish. Study information and consent processes were conducted during the farm business hours with the help of medically certified Spanish interpreters. Written informed consent was obtained from 173 participants. The protocol was approved by the Institutional Review Board at Augusta University. All measurements were performed on the morning of the health fair at the plant nursery at CL Farm. All methods were carried out in accordance with relevant guidelines and regulations.

### Handgrip strength measurement

Isometric handgrip strength was measured using a Jamar Hydraulic Hand Dynamometer (Jamar; Bolingbrook, IL). Grip strength was measured in both hands in a seated position with the arm at a 90-degree angle according to the National Health and Nutrition Examination Survey (NHANES) guidelines for hand dynamometry^16^. Handgrip strength was measured three times, and the highest score was used for each hand. The combined strength (in kilograms) of the right and left hands were used to create the absolute handgrip value. The relative handgrip was then calculated as the absolute handgrip divided by their BMI (kg/BMI)^6,12^. Handgrip strength asymmetry ratio is calculated as non-dominant/dominant handgrip strength. Those with handgrip strength ratio < 0.90 or > 1.10 have handgrip strength asymmetry.

### Anthropometry measurements

Height and weight were obtained according to standard procedures, using a wall-mounted stadiometer (Tanita Corporation of American, Arlington Heights, IL) and calibrated electronic scale (model CN2OL; Cardinal Detecto, Webb City, MO). BMI as weight (kg)/height (m^2^) was calculated using the CDC formula. Waist and hip circumferences (inches) were measured with minimal clothing at the level of umbilicus, and around the widest portion of the buttocks, respectively. The waist/hip circumference (W/Hip) ratio was calculated as the waist circumference divided by the hip circumference. After 5 min of rest, systolic (SBP) and diastolic blood pressures (DBP) were measured twice, each at least 1 min apart, in sitting position using manual mercury sphygmomanometer. The averages of two measurements were reported and used for analyses.

### Laboratory measurements

As previously described^26^, venous blood was collected after an overnight fast, and all blood samples were centrifuged immediately and stored at − 80 °C for analysis. Fasting plasma glucose (FPG) and lipid profile (total cholesterol, low-density lipoprotein cholesterol [LDL-cholesterol], high-density lipoprotein cholesterol [HDL-cholesterol], and triglycerides) were assessed by standard clinical laboratory methods at Premier Medical Laboratory Services (Greenville, SC). Fasting glucose was measured using hexokinase and glucose-6-phosphate dehydrogenase enzymatic method. Lipid parameters were measured by an enzymatic colorimetric method, using an automated analyzer (Cobas c 311/501 and Cobas c 502) and Cobas enzymatic reagents.

### Outcome variables

The outcome variables included BMI, LDL cholesterol, HDL cholesterol, cholesterol, triglycerides, BP, and blood glucose. In addition, these continuous variables were further classified to at risk or not using definitions from the American Diabetes Association (Table 1)^25^.

**Table 1:** Cutoff values of cardiometabolic risk factors.

Risk factor	Healthy range	At risk
Overweight/Obese	BMI between 19 and 25 kg/m^2^	BMI > 25 kg/m^2^
High LDL cholesterol	< 100 mg/dL	≥ 100 mg/dL
Low HDL cholesterol	> 60 mg/dL	≤ 60 mg/dL
High cholesterol	< 200 mg/dL	≥ 200 mg/dL
High triglycerides	< 150 mg/dL	≥ 150 mg/dL
High BP	< 120/80 mmHg	≥ 120/80 mmHg
High blood glucose	< 100 mg/dL	≥ 100 mg/dL

*BMI* body mass index, *BP* blood pressure, *LDL* low-density lipoprotein, *HDL* high-density lipoprotein.

### Statistical analysis

Normal distribution and homogeneity of variances were confirmed by Shapiro-Wilks *W* and Leven’s tests, respectively^27,28^. Partial Pearson’s correlation coefficients were used to examine the associations between absolute and relative handgrip as the independent variables and CMR factors as the dependent variables, with control for age. Similar statistical analyses were carried out in males and in females separately to investigate the sex-specific associations between handgrip strength and CMR factors. Absolute and relative handgrip strength measures were compared between each CMR factor at risk and not at risk using *t*-test. The cumulative associations of the composite CMR factors with absolute and relative handgrip measures were calculated using stepwise regression models, and backward-selection estimation was performed to select CMR factors correlated with handgrip strength with the backward selection at the significance level of 0.2 for removal from the model. Statistical analyses were carried out using SPSS software (version 23, IBM SPSS Statistics, Chicago, IL). A *P*-value < 0.05 was considered statistically significant.

## Results

### General characteristics of the participants

The population was composed of 173 Hispanic/Latino farmworkers (49% female) and the mean (± SEM) age was 35.1 ± 0.7 years. The clinical characteristics of the participants are presented in Table 2.

**Table 2:** Sex-specific clinical characteristics of the Hispanic farmworkers population.

	All	Males	Females	*P* value
N	173	88	85	
Age, years	35.1 ± 0.7	34.8 ± 1.0	35.4 ± 0.9	0.656
Absolute handgrip strength, kg	73.1 ± 1.6	89.2 ± 1.8	56.5 ± 1.9	< 0.001
Handgrip strength asymmetry, N	46 (27)	23 (26)	24 (28)	0.756
Handgrip strength asymmetry ratio	1.1 ± 0.1	1.1 ± 0.1	1.1 ± 0.1	0.406
Relative handgrip strength, kg/BMI	2.6 ± 0.1	3.3 ± 0.1	1.9 ± 0.1	< 0.001
BMI, kg/m^2^	28.8 ± 0.4	27.6 ± 0.4	30.1 ± 0.6	< 0.001
Waist circumference, inches	36.9 ± 0.3	36.9 ± 0.4	37.0 ± 0.5	0.890
W/Hip ratio	0.9 ± 0.1	0.9 ± 0.1	0.9 ± 0.1	0.035
FPG, mg/dL	100.4 ± 3.1	99.4 ± 3.7	101.4 ± 4.8	0.740
SBP, mm Hg	121.7 ± 1.2	126.9 ± 1.3	116.3 ± 1.4	< 0.001
DBP, mm Hg	77.2 ± 0.7	79.3 ± 1.0	75.0 ± 1.0	0.003
LDL-cholesterol, mg/dL	124.2 ± 2.5	131.4 ± 3.5	117.5 ± 3.4	0.005
HDL-cholesterol, mg/dL	47.8 ± 1.0	46.1 ± 1.4	49.47 ± 1.3	0.077
Total cholesterol, mg/dL	189.6 ± 2.9	196.2 ± 4.2	183.4 ± 3.9	0.027
Triglycerides, mg/dL	165.5 ± 7.6	187.8 ± 13.6	144.4 ± 6.6	0.005

Values are mean ± SEM.

*BMI* body mass index, *W/Hip* waist/hip circumference, *FPG* fasting blood glucose, *SBP* systolic blood pressure, *DBP* diastolic blood pressure, *LDL* low-density lipoprotein, *HDL* high-density lipoprotein.

### Variations in handgrip strength by participant characteristics

In the total sample, the means of absolute and relative handgrip strength were 73.1 ± 1.6 kg, 2.6 ± 0.1 kg/BMI, respectively. On average, absolute and relative handgrip strengths were 32.7 kg and 1.4 kg/BMI higher respectively in males than in females (*p* < 0.01, Table 2). Age was inversely correlated with absolute (*r* = − 0.17, *p* = 0.03) and relative handgrip strengths (*r* = − 0.28, *p* < 0.01). The differences in handgrip strength asymmetry and ratio between the male and the female were not statistically significant (*p* > 0.05).

### Associations between absolute handgrip strength and cardiometabolic risk factors

Supplementary Table S1 shows the partial correlations (adjusting for age) between absolute handgrip strength and CMR factors. A significant but weak inverse association was observed between absolute handgrip strength and triglycerides among males (*r* = − 0.25, *p* = 0.03). Likewise, there was a significant but weak inverse association between absolute handgrip strength and FPG among females (*r* = − 0.27, *p* = 0.01). Handgrip strength ratio was not correlated with any of the CMR factors in males or in females (*p* > 0.05).

### Associations between relative handgrip strength and cardiometabolic risk factors

Supplementary Table S2 reports the partial correlations (adjusting for age) between relative handgrip strength and CMR factors. In males, lower relative handgrip strength was significantly but weakly correlated with higher waist circumference (*r* = − 0.32, *p* < 0.01), W/Hip ratio (*r* = − 0.34, *p* < 0.01), and triglycerides (*r* = − 0.35, *p* < 0.01), while higher relative handgrip strength was significantly but weakly associated with higher HDL-cholesterol (*r* = 0.24, *p* = 0.04). In females, lower relative handgrip strength was significantly but weakly correlated with higher waist circumference (*r* = − 0.38, *p* < 0.01), and FPG (*r* = − 0.22, *p* < 0.01) (Supplementary Table S2). There were inverse trends of the correlations of relative handgrip with SBP and DBP among males and females, and an inverse trend for the correlation between relative handgrip and low-density lipoprotein among males only; however, none reached statistical significance.

### Comparisons of handgrip strength between cardiometabolic risk factor categories

Table 3 showed that males had lower absolute handgrip strength when their triglycerides levels were at risk (*p* = 0.021), and lower relative handgrip strength when their FPG (*p* = 0.034) and triglycerides (*p* = 0.002) levels were at risk. Females had lower relative handgrip strength when their FPG (*p* = 0.001) and BP (*p* = 0.004) were at risk.

**Table 3:** Comparisons of handgrip strength between cardiometabolic risk factor categories by sex.

	Absolute handgrip strength			Relative handgrip strength		
	At risk	Not at risk	P value	At risk	Not at risk	*P* value
**Male**						
BMI	87.8 ± 1.8	93.6 ± 5.1	0.180	–	–	–
FPG	88.2 ± 3.7	89.1 ± 1.8	0.812	3.0 ± 0.1	3.3 ± 0.1	0.034
BP	89.8 ± 2.2	87.1 ± 3.4	0.527	3.3 ± 0.1	3.3 ± 0.2	0.948
LDL-cholesterol	88.9 ± 1.9	88.7 ± 3.1	0.960	3.2 ± 0.1	3.3 ± 0.2	0.788
HDL-cholesterol	88.8 ± 1.8	89.5 ± 3.0	0.897	3.2 ± 0.1	3.6 ± 0.2	0.083
Total cholesterol	86.6 ± 2.4	90.8 ± 2.3	0.216	3.1 ± 0.1	3.4 ± 0.1	0.063
Triglycerides	84.8 ± 2.1	92.5 ± 2.4	0.021	3.0 ± 0.1	3.5 ± 0.1	0.002
**Female**						
BMI	56.8 ± 1.2	54.8 ± 2.4	0.508	–	–	–
FPG	53.9 ± 2.0	58.1 ± 1.3	0.085	1.7 ± 0.1	2.0 ± 0.1	0.001
BP	56.1 ± 1.9	56.9 ± 1.3	0.737	1.8 ± 0.1	2.0 ± 0.1	0.004
LDL-cholesterol	56.2 ± 1.2	58.0 ± 2.5	0.480	1.9 ± 0.1	2.0 ± 0.1	0.644
HDL-cholesterol	56.9 ± 1.3	55.2 ± 2.0	0.574	1.9 ± 0.1	1.9 ± 0.1	0.618
Total cholesterol	55.2 ± 2.1	57.2 ± 1.3	0.417	1.9 ± 0.1	1.9 ± 0.1	0.476
Triglycerides	56.4 ± 2.0	56.8 ± 1.3	0.837	1.9 ± 0.1	2.0 ± 0.1	0.266

Values are mean ± SEM.

*BMI* body mass index, *FPG* fasting blood glucose, *BP* blood pressure, *LDL* low-density lipoprotein, *HDL* high-density lipoprotein.

### Cumulative association of cardiometabolic risk factors with handgrip strength

The absolute handgrip strengths were explained by cumulative variations of age, BMI, and triglycerides (adjusted R-squared = 0.09) in males, and by FPG and SBP (adjusted R-squared = 0.11) in females (Table 4). The relative handgrip strengths were explained by cumulative variations of FPG, LDL-cholesterol, total cholesterol, and triglycerides (adjusted R-squared = 0.17) in males, and by age, FPG, DBP, LDL-cholesterol, total cholesterol, and triglycerides (adjusted R-squared = 0.13) in females (Table 4).

**Table 4:** Cumulative association of cardiometabolic risk factors with handgrip strength by sex.

	Absolute handgrip strength		Relative handgrip strength	
	β (95% CI)	P value	β (95% CI)*	P value
**Male**	Adjusted R-squared = 0.09		Adjusted R-squared = 0.17	
Age	− 0.35 (− 0.69, − 0.01)	0.042		
BMI	0.68 (− 0.15, 1.52)	0.107		
FPG			− 0.38 (− 0.83, 0.07)	0.097
LDL-cholesterol			− 1.40 (− 2.55, − 0.24)	0.018
Total cholesterol			0.82 (− 0.22, 1.87)	0.121
Triglycerides	− 0.03 (− 0.06, − 0.00)	0.024	− 0.20 (− 0.36, − 0.04)	0.014
**Female**	Adjusted R-squared = 0.11		Adjusted R-squared = 0.13	
Age			− 1.00 (− 2.14, 0.15)	0.086
FPG	− 0.08 (− 0.13, − 0.03)	0.002	− 0.22 (− 0.46, 0.01)	0.059
SBP	0.16 (− 0.00, 0.33)	0.056		
DBP			− 0.79 (− 1.77, 0.19)	0.114
LDL-cholesterol			− 0.59 (− 1.39, 0.20)	0.142
Total cholesterol			0.61 (− 0.09, 1.31)	0.086
Triglycerides			− 0.14 (− 0.30, 0.03)	0.102

*BMI* body mass index, *FPG* fasting blood glucose, *SBP* systolic blood pressure, *DBP* diastolic blood pressure, *LDL* low-density lipoprotein.

*β (95% CI) were multiplied by 100.

## Discussion

To our knowledge, this is the first study to characterize the values of absolute and relative handgrip strengths and to investigate their associations with CMR factors in a Hispanic/Latino migrant farmworker population. To date, mean values of absolute and relative handgrip strengths have not been studied in the Hispanic adult population in general, and especially Hispanic/Latino migrant farmworkers. Data from 2011–2012 NHANES showed that the means of absolute and relative handgrip strengths in the U.S. adult population were similar to our Hispanic farmworkers in both sexes^16^. In males, the combined absolute handgrip strengths reported in the NHANES population in comparison with our study participants were 89.7 ± 0.8 kg and 89.2 ± 1.8 kg, respectively. The relative handgrip strength in the NHANES and our population were 3.2 ± 0.1 kg/BMI and 3.3 ± 0.1 kg/BMI, respectively. In females, the combined absolute handgrip strength in NHANES and our population were 56.1 ± 0.5 kg and 56.5 ± 1.9 kg, respectively. The means of relative handgrip strength in NHANES and our population were 2.0 ± 0.02 kg/BMI and 1.9 ± 0.1 kg/BMI, respectively. Multiple studies have shown that handgrip strength was relatively stable from 20–59 years, but decreased from 60 to 79 years^29,30^. The average ages of our population (35.1 ± 0.7 years) and NHANES population (47.5 ± 0.8 years) were both below age 50, which might explain the similar finding of absolute and relative handgrip strength in both populations.

This study found that in Hispanic/Latino farmworkers, lower handgrip strength is correlated with greater CMR factors in a sex-specific manner. In both males and females, relative handgrip strength was coupled with obesity and abdominal obesity markers, which is consistent with previous research^31^ indicating that obesity is associated with decreased muscle activity and strength. Higher relative handgrip strength was associated with lower waist circumference and W/Hip ratio in males, and lower waist circumference in females. A large population study found that grip strength was inversely associated with all-cause mortality, myocardial infarction, and stroke, and was a stronger predictor of all-cause and cardiovascular mortality than SBP^32^.

It has been postulated that the underlying mechanisms which could explain the relationship between obesity and reduced handgrip strength are inflammation and insulin resistance^33–35^. First, in the state of obesity, the inflammatory responses are activated through the production of proinflammatory cytokines and adipokines from adipose tissue^36,37^. Chronic inflammation and increased oxidative stress could contribute to the decline of muscle mass and strength^38,39^. Second, obesity is linked to insulin resistance, and the most crucial factor in modifying insulin sensitivity is the release of non-esterified fatty acids which diminish insulin receptor signaling^40^. Also, decreased insulin secretion due to the defect of the β-cell in the pancreas could explain the association between obesity and insulin resistance^35^. Barzilay et al. observed that insulin resistance could be related to the reduction in muscle strength of quadriceps muscle in elderly^41^. A possible physiological mechanism underlying diabetes and handgrip strength may be that the decreased muscle mass can lead to less efficient glucose uptake, which stimulates insulin resistance that can progress to diabetes^23^. Abbatecola et al. did not only find that insulin resistance was not linked to lower handgrip strength, but also observed that this relationship was significant in women while it was not significant in men, which is consistent with our finding^42^. However, other previous research indicated that handgrip strength was associated with dysglycemia^43^, prediabetes^23^, and type 2 diabetes in males^19^. It has been postulated that the difference in muscle strength between males and females from early adulthood may elucidate the difference in the association between handgrip strength and FPG^44^.

The lipid profile was also associated with handgrip strength for males. The inverse correlation between relative handgrip strength and HDL-cholesterol and triglycerides is comparable to the results of previous research that CVD risk was associated with lower handgrip in both male and female participants^16^. Other research supports that greater muscle strength affects the levels of lipoproteins^45,46^. However, we did not find the same relationship in females in our population. It may be plausible that the variation in the association between handgrip strength and CMR factors among both male and female participants may be due to differences in sex hormones^47,48^. A study by Michael and colleagues found that hormone therapy in postmenopausal women did not enhance their handgrip strength^49^, while a study by Page and colleagues showed that the administration of exogenous testosterone to older men enhanced their handgrip strength^50^. Future studies with larger sample sizes are warranted to unravel the underlying mechanism for these sex differences.

Limitations of the study should be acknowledged. This study is observational, which does not provide a causal explanation for the findings. Also, the current study has a relatively small sample size and only one single measure of muscle strength. Third, our participants were relatively young, and most of them were in their young adulthood. Therefore, our results of the relationship between handgrip strength and CMR factors cannot be generalized to other age groups. Similarly, only Hispanic/Latino farmworkers were included in this analysis, thus the appliance of our results to other race groups needs caution. Lastly, some relevant confounders were not collected in this study, such as dietary intake, physical activity, socioeconomic status, and family history.

## Conclusions

In conclusion, this is the first study to report the values of absolute and the relative handgrip strengths, and their associations with sex-specific CMR factors among the Hispanic/Latino farmworkers. Handgrip strength is not only a marker of whole-body strength but also appears to be an indicator of CMR. As such, routine testing of handgrip strength should be considered when assessing the CMR of Hispanics in the clinical setting. Further research is required for studying potential underlying mechanisms.

## Supplementary information

Supplementary Information.
