# Supplementary material for: Handgrip and sex-specific cardiometabolic risk factors in Hispanic/Latino migrant farmworkers
Source: Sci Rep. 2021 May 13;11:10272. doi: 10.1038/s41598-021-89138-y (PMC8119492; doi:10.1038/s41598-021-89138-y)
Supplement: Supplementary file 1 — Supplementary Information. [file 41598_2021_89138_MOESM1_ESM.docx]

### **Supplementary Tables**

### **Table S1. Correlations of absolute isometric handgrip strength and cardiometabolic risk factors in Hispanic farmworkers by sex**

|  | ***Males*** | | ***Females*** | |
| --- | --- | --- | --- | --- |
|  | *r* | *P* value | *r* | *P* value |
| BMI, kg/m^2^ | 0.02 | 0.87 | 0.17 | 0.13 |
| Waist circumference, inches | 0.18 | 0.09 | 0.17 | 0.13 |
| W/Hip ratio | -0.12 | 0.26 | -0.12 | 0.26 |
| FPG, mg/dL | -0.06 | 0.59 | -0.27 | 0.01 |
| SBP, mm Hg | 0.14 | 0.21 | 0.18 | 0.11 |
| DBP, mm Hg | 0.21 | 0.06 | 0.12 | 0.27 |
| LDL-cholesterol, mg/dL | -0.09 | 0.44 | 0.00 | 1.00 |
| HDL-cholesterol, mg/dL | 0.08 | 0.51 | 0.01 | 0.92 |
| Total cholesterol, mg/dL | -0.14 | 0.25 | 0.01 | 0.91 |
| Triglycerides, mg/dL | -0.25 | 0.03 | -0.03 | 0.82 |

Note: Relationships are adjusted for age. Abbreviations: BMI, body mass index; W/Hip, waist/hip circumference; FPG, fasting blood glucose; SBP, systolic blood pressure; DBP, diastolic blood pressure; LDL, low-density lipoprotein; HDL, high-density lipoprotein.

### **Table S2. Correlations of relative handgrip strength and cardiometabolic risk factors in Hispanic farmworkers by sex**

|  | ***Males*** | | ***Females*** | |
| --- | --- | --- | --- | --- |
|  | *r* | *P* value | *r* | *P* value |
| Waist circumference, inches | -0.32 | <0.01 | -0.38 | <0.01 |
| W/Hip ratio | -0.34 | <0.01 | -0.15 | 0.19 |
| FPG, mg/dL | -0.20 | 0.09 | -0.22 | <0.01 |
| SBP, mm Hg | -0.06 | 0.55 | -0.06 | 0.61 |
| DBP, mm Hg | -0.01 | 0.95 | -0.13 | 0.24 |
| LDL-cholesterol, mg/dL | -0.23 | 0.05 | 0.04 | 0.76 |
| HDL-cholesterol, mg/dL | 0.24 | 0.04 | 0.10 | 0.37 |
| Total cholesterol, mg/dL | -0.22 | 0.06 | 0.06 | 0.61 |
| Triglycerides, mg/dL | -0.35 | <0.01 | -0.13 | 0.24 |

Note: Relationships are adjusted for age. Abbreviations: W/Hip, waist/hip circumference; FPG, fasting blood glucose; SBP, systolic blood pressure; DBP, diastolic blood pressure; LDL, low-density lipoprotein; HDL, high-density lipoprotein.
